# An interpretable progressive residual network for automated multiclass diabetes diagnosis

**DOI:** 10.1038/s41598-026-51603-x

**Published:** 2026-05-04

**Authors:** Huaxin Fan, Zhendong Li, Ning Yan, Hao Liu

**Affiliations:** 1 School of Information Engineering, Ningxia University, Yinchuan, China; 2 Heart Centre, Department of Cardiovascular Diseases, General Hospital of Ningxia Medical University, Yinchuan, China

**Keywords:** diabetes mellitus, pre-diabetic stage, deep learning, interpretability, progressive residual network, risk stratification, Biomarkers, Computational biology and bioinformatics, Diseases, Medical research

## Abstract

Diabetes mellitus remains one of the most widespread and burdensome chronic diseases worldwide, yet invasive assays and high costs constrain early detection. Existing machine-learning studies often reduce diagnosis to a binary task and overlook the clinically important pre-diabetic stage; additionally, many deep models act as uninterpretable “black boxes”. To address these gaps, we propose ProgMDD, an interpretable progressive residual network for multiclass diabetes diagnosis using routine clinical biomarkers. Employing a strict, leakage-free pipeline, LASSO-based feature selection and resampling were applied exclusively to the training set, yielding a compact, robust input panel. After comparing PCA, t-SNE, and UMAP, we selected UMAP for visualization because it optimally balances global and local structure to illustrate progressive class separation. ProgMDD integrates a progressive residual architecture with channel attention and multi-level regularization to enhance feature learning. Rigorously compared against multiple baselines, ProgMDD achieved 97.02% mean accuracy under 5-fold cross-validation, reinforced by a 97.59% accuracy on the purely original, imbalanced hold-out test set and supported by multiple ablation studies. The concordance between LASSO and SHAP rankings supports biological plausibility and model transparency. By uniting interpretable deep learning with low-cost clinical data, ProgMDD furnishes a feasible approach for early screening and risk stratification in primary care, providing a transferable methodological paradigm for other chronic-disease prediction tasks.

**Supplementary Information:**

The online version contains supplementary material available at 10.1038/s41598-026-51603-x.

## Introduction

Diabetes mellitus is a chronic endocrine disorder characterized by the body’s inability to properly regulate blood glucose levels^1^. It has become one of the most prevalent and serious chronic diseases worldwide. In 2021, diabetes contributed to an estimated 6.7 million deaths, and by 2022, approximately one in ten people globally had diabetes–a figure projected to rise from 537 million in 2021 to 783 million by 2045 due to population aging, urbanization, and increasingly sedentary lifestyles and unhealthy diets^2^. This disease also imposes a tremendous economic burden. In the United States, the total cost of diagnosed diabetes was estimated at $412.9 billion in 2022^3^, accounting for roughly one in every four health care dollars. On average, individuals with diabetes incur about 2.6 times higher medical expenditures than those without the disease^3^, reflecting its substantial medical and societal impact.

Despite this widespread burden, nearly half of type 2 diabetes (T2D) cases worldwide remain undiagnosed^4^. As a result, these individuals do not receive timely treatment or preventive measures to avoid or delay diabetes-related complications, and undiagnosed T2D is associated with a higher risk of mortality compared to normoglycemic individuals^5^; this is partly because roughly one-third of T2D patients show no symptoms until after complications have developed. Undiagnosed cases also impose a hidden financial strain, costing an estimated $4,250 per person per year in the United States^6^ and contributing to preventable health care expenditures. However, current screening and diagnostic approaches still rely on invasive blood-based biochemical assays, such as fasting plasma glucose (FPG) and glycated hemoglobin (HbA1c) measurements, or an oral glucose tolerance test (OGTT), which require clinical analyzers or spectrophotometric instruments^7^. While well-established, these methods demand costly laboratory infrastructure and trained personnel, often involve fasting or repeated blood sampling, and are time-consuming, making large-scale screening difficult to implement–especially in resource-limited settings^7^. These limitations underscore the need for more accessible, cost-effective, and reliable strategies for early diabetes detection and diagnosis.

With the rapid advancement of digital technologies and artificial intelligence, substantial computational efforts have been devoted to integrating machine learning algorithms into diabetes research to assist clinicians in making efficient and data-driven diagnostic decisions. Neural network (NN)-based algorithms, such as multi-layer perceptrons (MLP)^8^, deep neural networks (DNN)^9^, and conventional machine learning (CML) models, have shown promising results^10^. For instance, the study in^11^ proposed a deep learning approach that transforms numerical features into image representations, enabling CNNs to improve robustness and diagnostic accuracy in diabetes detection. Moreover, another study^12^ proposed a deep neural network enhanced with PCA and optimized using the Gray Wolf Optimization (GWO) algorithm to predict diabetes, achieving superior performance compared to SVM, Naïve Bayes (NB), decision tree (DT), and XGBoost models, with an overall accuracy of 96%. Although numerous studies have focused on early-stage diabetes prediction, most existing approaches have been limited to binary classification, distinguishing only between diabetic and non-diabetic individuals, while neglecting the pre-diabetic stage that represents the critical transition from normal to diabetic conditions. This omission is crucial, as once diabetes is clinically diagnosed, patients often face lifelong treatment and considerable economic burden, resulting in irreversible health and financial consequences.

Although artificial intelligence has proven highly effective in critical applications such as disease diagnosis^13^, its decision-making process often remains a “black box.” This lack of transparency limits our understanding of internal algorithmic mechanisms and hinders clinical trust, particularly in high-stakes medical scenarios. To bridge this gap, we propose ProgMDD, an interpretable progressive residual network with channel attention, explicitly tailored for the multiclass diagnosis of diabetes (Normal, Prediabetes, and Diabetes). To ensure maximum methodological rigor and eliminate any risk of data leakage, we established a strict, real-world evaluation pipeline. Specifically, the original clinical dataset is partitioned prior to any preprocessing. Subsequently, the Least Absolute Shrinkage and Selection Operator (LASSO) algorithm and SMOTE-Tomek resampling are applied exclusively within each training fold during cross-validation. This robust strategy successfully mitigates severe class imbalance and isolates the six most discriminative clinical biomarkers without compromising the integrity of the unseen validation and test sets.

To substantiate the architectural superiority and clinical reliability of ProgMDD, we conducted comprehensive evaluations on the original, imbalanced hold-out test set, supplemented by rigorous 5-fold cross-validation. The proposed framework was extensively benchmarked against a diverse suite of order-agnostic baseline models, including Multinomial Logistic Regression (L1), SVM, Random Forests, XGBoost, a basic MLP, and a clinically intuitive Rule-based Decision Tree. Furthermore, to explicitly justify the application of 1D convolutions on tabular data, we performed detailed ablation studies and feature permutation sensitivity analyses, empirically demonstrating that ProgMDD’s performance stems from weight-sharing regularization and dynamic feature recalibration rather than arbitrary variable ordering. For interpretability, Uniform Manifold Approximation and Projection (UMAP) was utilized to visualize latent class separability, while Shapley Additive Explanations (SHAP) quantified the precise contribution of each biomarker. By harmonizing strict methodological safeguards, exhaustive empirical validation, and highly transparent decision-making, ProgMDD provides a theoretically sound and computationally efficient framework for large-scale, early multiclass diabetes screening.

## Related works

Accurate early diagnosis of diabetes remains a major clinical challenge^14^. While biomarker-based assays are routine, interpreting the transitional metabolic profiles, particularly distinguishing the ambiguous “Pre-diabetic” state from healthy or overtly diabetic states, requires robust analytical tools^15^. Recently, data-driven approaches have significantly advanced automated diabetes screening^16^. However, to translate these models into primary care, research must address specific challenges inherent to clinical tabular data: multiclass stratification, severe class imbalance, feature optimization, and clinical interpretability.

### The challenge of multiclass stratification and prediabetes

A substantial portion of existing literature focuses primarily on binary classification (e.g., Healthy vs. Diabetic). Numerous studies have successfully applied machine learning and ensemble models, such as Random Forests and XGBoost, to predict binary diabetes onset with high accuracy^17–19^. For instance, Lee et al.^20^ and Zhou et al.^21^ demonstrated the superiority of ensemble learning in identifying high-risk diabetic cohorts. However, in real-world clinical settings, diabetes progression is a continuum. The transitional “Pre-diabetic” stage represents a critical window for intervention, yet it is notoriously difficult to classify due to overlapping biomarker distributions with other classes. While some studies^22^ have attempted to classify this rare intermediate class using standard algorithms, standard models often struggle to capture the subtle, non-linear decision boundaries required for accurate multiclass stratification without losing predictive precision.

### Handling class imbalance in clinical tabular data

Clinical datasets for diabetes screening are inherently imbalanced, with pre-diabetic cases typically underrepresented. To mitigate this, researchers have adopted various data-level and algorithm-level interventions. Sadeghi et al.^22^ and Talebi et al.^23^ utilized threshold adjustments and cost-sensitive learning to improve the prediction of rare diabetes classes. Other approaches, such as the stacking ensemble developed by Elgendy et al.^24^ and Kibria et al.^25^, incorporated oversampling techniques like SMOTE and SMOTE-Tomek to balance the data distributions before training. While these resampling methods are effective for traditional machine learning, applying them directly to deep neural networks often leads to severe overfitting, as deep networks tend to memorize the synthetic minority samples rather than learning generalized physiological patterns.

### Feature optimization in deep learning for tabular data

Deep learning (DL) is increasingly utilized for diabetes prediction due to its capacity for complex representation learning^26,27^. However, tabular clinical data differs fundamentally from images or text; it often contains a mix of highly discriminative features (e.g., HbA1c, BMI) and noisy, low-impact variables. Standard DL architectures, such as basic MLP or 1D-CNNs, treat all input features equally across spatial dimensions. While advanced models like DeepNetX2^27^ utilize external correlation-based wrappers for feature selection, there remains a need for internal architectural mechanisms that can adaptively weight and prioritize dominant clinical biomarkers during the training process itself, without discarding potentially useful secondary indicators.

### Explainability and clinical trust

The “black-box” nature of complex models creates substantial barriers to clinical adoption. Explainable Artificial Intelligence (XAI) frameworks, particularly SHAP and LIME, have been widely implemented to provide prediction rationales. Studies by Mohanty et al.^28^, Tasin et al.^29^, and Chen et al.^30^ successfully integrated SHAP into ensemble and DL models to identify influential features. However, in many tabular DL applications, XAI is treated merely as a post-hoc add-on. For an algorithm to be trusted as a screening tool, its feature attributions must not only be internally consistent but also transparently align with established medical guidelines (such as the ADA standards). Triangulating non-linear deep learning explanations with transparent statistical methods (like LASSO) remains an underexplored area in diabetes prediction.

### Unresolved gaps and the proposed ProgMDD framework

Despite the rapid advancements in automated diabetes prediction, a critical gap remains: the lack of a unified deep learning framework explicitly designed to handle the overfitting risks of imbalanced multiclass tabular data while offering reliable, guideline-aligned interpretability. To address these specific limitations, this study proposes ProgMDD. Specifically, our model introduces targeted solutions for each unresolved challenge:

1. To address multiclass imbalance and overfitting: We integrate SMOTE-Tomek with a progressive residual structure. This architectural novelty dynamically increases regularization in deeper layers, effectively preventing the network from memorizing synthetic minority samples in the “Pre-diabetic” class.
2. To optimize tabular feature learning: We incorporate a channel attention mechanism that adaptively recalibrates feature weights, allowing the model to internally focus on high-impact routine biomarkers (like HbA1c).
3. To ensure clinical trust: We employ a dual-interpretability framework, using both SHAP and LASSO, to mutually validate that the model’s internal decision logic strictly aligns with established clinical pathogenesis.

## Data collection

The dataset utilized in this study was acquired from the Laboratory of Medical City Hospital, Iraq^31^. It contains clinical information from Iraqi patients across three distinct classes: Diabetic (844 instances), Non-Diabetic (103 instances), and Pre-Diabetic (53 instances). During the initial data cleaning phase, we removed 170 duplicate records and corrected 5 records with format inconsistencies. It is important to clarify that “duplicate” here refers to literal duplicate entries (i.e., exact row matches across all features and labels). Because the original public dataset lacks unique patient identifiers and visit timestamps, we could not distinguish between repeated visits from the same patient and accidental data entry errors; thus, all exact duplicates were removed. Furthermore, the 5 format inconsistencies only involved standardizing text strings (e.g., stripping trailing spaces from labels like “Y " or “N " to “Y” and “N”). We did not perform any clinical re-labeling based on medical rules, and all target labels preserve the original ground truth provided by the dataset. For specific details, please refer to Table 1. Noticeably, the dataset exhibits a severe natural class imbalance, with a ratio of approximately 16:2:1 (Diabetic: Non-Diabetic: Pre-Diabetic). This imbalance and the presence of noise necessitated the multi-stage preprocessing and nested balancing pipeline detailed in the Method.

**Table 1:** Overview of the original dataset.

Feature	Description	Type	Value Range	Class Distribution
Gender	0 = Female,1 = Male	Categorical	0/1	Y: 844 (84.4%)
AGE	Age of the subject in years	Numerical	20–79	N: 103 (10.3%)
Urea	A measurement of urea (in mg/dL) in the blood	Numerical	0.5–38.9	P: 53 (5.3%)
Cr (Creatinine)	Measures the level of creatinine in the blood (mg/dL).	Numerical	6.0–800.0	Total: 1000
HbA1c (Glycated Hemoglobin)	A key indicator of average blood glucose levels over the past 2–3 months.	Numerical	0.9–16.0	
Chol (Cholesterol)	Total cholesterol in the blood (mg/dL).	Numerical	0.0–10.3	
TG (Triglycerides)	Measures the amount of fat in the blood (mg/dL)	Numerical	0.3–13.8	
HDL (High-Density Lipoprotein)	The “good” cholesterol (mg/dL)	Numerical	0.2–9.9	
LDL (Low-Density Lipoprotein)	The “bad” cholesterol (mg/dL)	Numerical	0.3–9.9	
VLDL (Very Low-Density Lipoprotein)	Another type of “bad” cholesterol (mg/dL) carries triglycerides	Numerical	0.1–35.0	
BMI (Body Mass Index)	A measure of body fat based on height and weight (kg/m²).	Numerical	19.0–47.8	
Class	Target label	Categorical	Y/N/P	

Description of initial preprocessing: After removing and correcting duplicate and mislabeled subjects from the original dataset, 694 diabetic, 96 non-diabetic, and 40 predicted-diabetic subjects remained, with a total of 830 cases.

## Method

### Overall framework

**Fig. 1:**
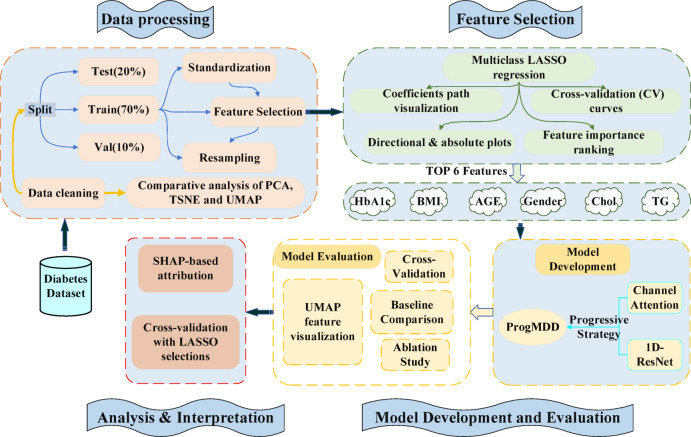
Overall research framework. Detailed workflow illustrating data preprocessing, feature selection, model development, evaluation, and interpretability analysis.

The proposed framework (Fig. 1) is designed as a strictly nested pipeline to ensure the scientific integrity of the results and prevent any form of data leakage. The workflow comprises the following five stages:

Stage 1: Data Partitioning and Preprocessing. To maintain strict independence, we first perform initial cleaning on the raw dataset and immediately partition it into stratified training (70%), validation (10%), and independent test sets (20%). Crucially, all subsequent parameters for standardization and feature selection are derived solely from the training partition to ensure the validation and test sets remain entirely representative of real-world clinical distributions.

Stage 2: Within-Train Feature Engineering. On the training set only, we apply Multiclass LASSO to identify a stable subset of predictive features (e.g., HbA1c, BMI, AGE). Following selection, the SMOTE-Tomek pipeline is applied exclusively to the training samples to mitigate class imbalance, while the evaluation sets remain in their original, unaugmented state.

Stage 3: Model Development and Progressive Learning. We implement the ProgMDD architecture, integrating progressive regularization with channel attention into a 1D-ResNet backbone. The model is optimized using the AdamW optimizer with a OneCycleLR scheduler. Training dynamics are monitored via UMAP-based feature visualization of the internal layers (e.g., relu_fc1) to ensure the classes become progressively distinct.

Stage 4: Multi-level Evaluation and Comparison. To rigorously validate the proposed architecture, we conduct a three-tier evaluation:

- Cross-Validation: Performing a stratified 5-fold cross-validation to ensure statistical stability and generalizability.
- Baseline Comparison: Comparing ProgMDD against standard machine learning and deep learning models to demonstrate superior multiclass stratification.
- Ablation Study: Systematically removing the progressive strategy and channel attention modules to quantify their individual contributions to preventing overfitting and enhancing feature focus.

Stage 5: Clinical Interpretation. Finally, we utilize a dual-interpretability framework (SHAP and LASSO) to identify key biochemical indicators and validate the model’s decision logic against established clinical knowledge.

### Data processing

The raw dataset was fully de-identified to ensure participant privacy. The authors had no access to any participant-identifiable information during or after data collection. The initial dataset contained 1,000 records across three diagnostic classes. To ensure data quality, we first performed basic cleaning and feature pruning by retaining 11 key biological indicators (Gender, Age, Urea, Cr, HbA1c, Chol, TG, HDL, LDL, VLDL, and BMI) together with the class label according to^32^, and removing non-informative identifiers (ID, No_Pation). Gender was encoded numerically (M = 1, F = 0), and all duplicate instances were removed to minimize overfitting risks. Labels were standardized into three categories (Y, N, P).

To prevent data leakage and ensure a rigorous evaluation, we implemented a strictly nested preprocessing pipeline. We first partitioned the cleaned real dataset into a training set (70%), a validation set (10%), and a test set (20%) using stratified sampling. All subsequent operations, including standardization, feature selection, resampling, and hyperparameter tuning, were performed exclusively within the training portion of each fold in cross-validation.

Feature Standardization: We calculated the column-wise means and standard deviations solely from the training set. We then used these statistics to scale all three subsets. This ensures the model evaluates “unseen” data without prior knowledge of the test distribution.

Feature Selection and Tuning: Feature selection via LASSO (detailed in Section Feature Selection) was conducted strictly on the training partition. Similarly, all hyperparameter tuning was restricted to the training phase to avoid optimistic bias.

Data Augmentation: To mitigate class imbalance, the SMOTE-Tomek Links pipeline was applied only to the training set. We generated synthetic samples using Eq. (1), where $X_i$ denotes a minority-class sample, $X_{zi}$ its k-nearest neighbor, and $rand \in [ 0,1]$ a random scalar. The neighbor parameter *k* was set adaptively according to Eq. (2) to preserve minority-graph connectivity. Subsequently, Tomek Links removed cross-class nearest-neighbor pairs to purify the decision boundary. The validation and test sets remained in their original, unaugmented distribution to reflect real-world clinical scenarios.

1 $$x_{new}= x_i + rand ( x_{zi}-xi)$$

2 $$k=min(5,4_{min} - 1)$$

Finally, the standardized feature matrix was reshaped into a 3D format (batch × channel × length). This format aligns with our 1D-ResNet architecture. Class labels were converted to integer indices for the multiclass cross-entropy loss calculation.

### Dimensionality reduction analysis

Dimensionality reduction and visualization are essential for exposing a dataset’s core structure and guiding subsequent modeling^33^. In biomedical research, linear approaches like PCA and nonlinear embeddings such as t-SNE and UMAP are widely adopted^34^.

PCA is extensively employed for clinical data analysis by projecting variables onto orthogonal principal components to capture dominant variance^35^. However, its linearity assumption may yield unsatisfactory results when biomarkers exhibit complex, nonlinear correlations, as is often the case in diabetes datasets. To address these complexities, t-SNE provides intuitive nonlinear visualization by modeling pairwise similarities to expose local clusters^36^. Nevertheless, t-SNE tends to prioritize local details at the expense of global structure and can be computationally intensive.

To balance global and local structures while maintaining computational efficiency, we adopted UMAP^37^. Based on manifold theory and topological data analysis, UMAP constructs a weighted high-dimensional graph to capture local relationships and optimizes a low-dimensional embedding via stochastic gradient descent. Unlike t-SNE, UMAP preserves more global connectivity while revealing local groupings^38^. These properties, combined with its stable visualization performance, make UMAP the superior choice for analyzing the intricate patterns in diabetes clinical indicators.

### Feature selection

In this phase, we performed a methodical analysis to identify predictors most relevant to diabetes outcomes. Following the nested pipeline described in Data Processing, feature selection was conducted exclusively on the training partition after data standardization. This approach ensures that the selection process remains independent of the test data. Feature selection plays a key role in simplifying the model and enhancing interpretability. Among various techniques, LASSO is a powerful method for variable selection and regularization. It has recently gained importance in predicting quality-related outcomes in healthcare^39^. The core idea of LASSO is to add an L1 regularization term to the loss function. This leads to the objective function shown in Eq. (3).

3 $$\mathop{min}_{\beta} \frac{1}{2n} ||y-X\beta ||^2_2 + \lambda ||\beta ||_1$$

In Eq. (3), $y \in \mathcal{R}^n$ is the response vector, $X\in \mathcal{R}^{n\times p}$ is the design matrix of predictors, $\beta \in \mathcal{R}^p$ is the coefficient vector, *n* is the sample size, and $\lambda \geq 0$ controls the strength of the L1 penalty. This penalty shrinks coefficients to reduce model complexity and prevent overfitting. It also performs automatic feature selection by driving the coefficients of irrelevant variables exactly to zero.

In this study, we applied LASSO feature selection by leveraging the principle of L1-induced sparsity. We incorporated an L1 penalty into multinomial logistic regression so that statistically redundant or weak predictors received coefficients of zero, yielding a compact and interpretable feature subset without sacrificing discriminative power. For implementation, we explored a logarithmic grid of $\lambda$ values to control model complexity. K-fold cross-validation with log-loss guided the choice of the penalty strength, using the relationship $C=1/\lambda$ for equivalence. The cross-validation error for a given $\lambda$ was computed as Eq. (4), where *k* is the number of folds, $J(\cdot)$ is the log-loss, $y_i$ is the observed outcome in the *i*-th fold, and $\hat{y}_i (\lambda)$ is the prediction obtained with penalty $\lambda$.

4 $$CV(\lambda) = \frac{1}{k} \sum_{i=1}^k L (y_i, \hat{y}_i(\lambda))$$

At each $\lambda$, we obtained a coefficient matrix with dimensions “number of classes × number of features.” For each feature, we recorded the maximum absolute coefficient across classes as its representative strength. We plotted coefficient paths, tracked the number of non-zero features as $\lambda$ varied, and computed the cross-validation error curve to identify $\lambda.min$ (minimum mean log-loss) and $\lambda 1se$ (the simplest model within one standard deviation of the minimum error).

Based on these results, we defined features with absolute coefficients greater than 1e-5 in any class as selected. We ranked them by both directional and absolute importance to form a robust candidate set ($\lambda.min$) and a more parsimonious alternative set ($\lambda 1se$). The selected features were then used to train downstream deep models. By restricting the input channels to variables retained by LASSO, we reduced redundancy and improved generalization. This linear, interpretable screening also provided stable feature candidates and directional priors for subsequent SHAP-based interpretation.

### Design of ProgMDD

**Fig. 2:**
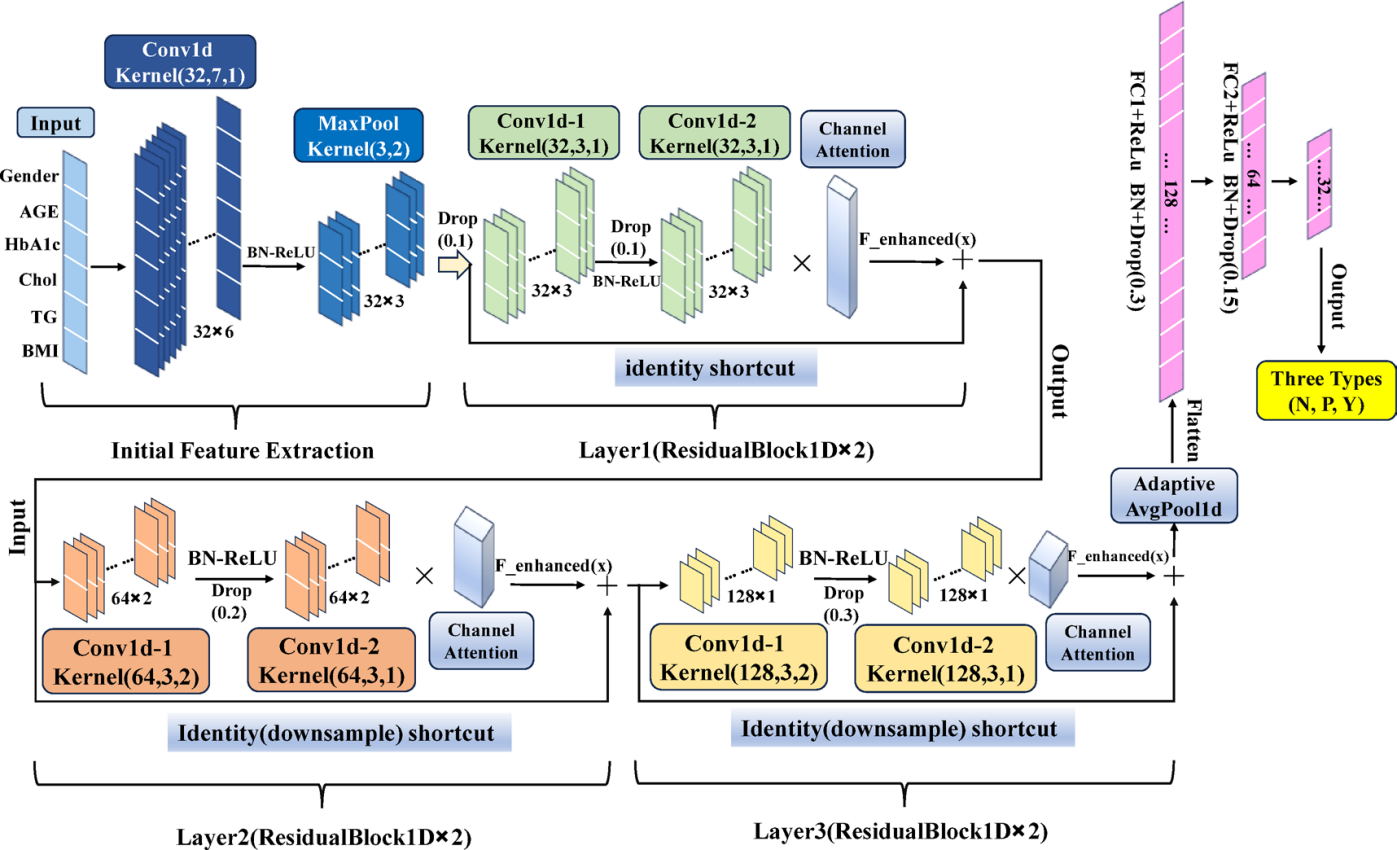
Architecture of ProgMDD. The network comprises an initial Conv1D layer, three progressive residual layers (each with two blocks featuring batch normalization, ReLU, progressive dropout, and channel attention), and a final classifier with global average pooling and two fully connected layers. Subsequent residual layers implement downsampling in their initial blocks.

Owing to its superior ability to model nonlinear relationships end‑to‑end^40^, deep learning is widely applied to biomedical prediction tasks. While standard tabular biomedical indicators (e.g., AGE, BMI, HbA1c) do not inherently possess strict spatial adjacency, organizing them as a one-dimensional sequence allows 1D convolutions to act as highly regularized feature extractors. Unlike standard MLPs, which are prone to severe overfitting on limited tabular data, the weight-sharing mechanism of 1D-CNNs provides robust regularization against noise. Furthermore, any potential bias introduced by the arbitrary sequential ordering of tabular features is explicitly mitigated by the subsequent Channel Attention mechanism, which globally recalibrates feature importance regardless of their initial spatial positions. However, plain 1D-CNNs tend to exhibit performance degradation as network depth increases. Therefore, we propose a progressive 1D-ResNet with channel attention to discriminate diabetes types, adopting a progressive strategy to expand the receptive field and feature hierarchy. The overall architecture of our proposed ProgMDD is shown in Fig. 2.

Specifically, to address the inherent challenges of small-scale, high-noise clinical datasets, ProgMDD introduces significant architectural improvements over standard ResNet designs. Standard ResNets, originally optimized for large-scale, high-dimensional image corpora, often suffer from severe overfitting and unstable gradients when directly applied to low-dimensional biomedical sequences. To overcome this, ProgMDD departs from uniform layer configurations and implements a “Progressive Strategy” that manifests in hierarchical feature construction and dynamic regularization. Unlike standard residual architectures, ProgMDD systematically expands the receptive field while concurrently intensifying regularization constraints. In the early stages, the network utilizes wide convolutions with low dropout rates to safely capture fine-grained, high-resolution local details without losing information. As the network deepens and features become more abstract, the progressive regularization mechanism kicks in: dropout rates explicitly increase in deeper stages, and the classifier compresses the feature dimensions. What progressive regularization contributes in terms of learning capability is crucial: it effectively balances bias and variance. By penalizing deep, complex representations more heavily than shallow ones, it prevents the model from memorizing synthetic noise or minor distribution shifts. Furthermore, training employs progressive optimization strategies as well: a one-cycle learning rate schedule (warmup-peak-decay) accelerates early convergence, while an exponential moving average (EMA) of parameters smooths the later-stage learning dynamics. This tailored combination of progressive dropout, dynamic learning rates, and EMA provides a highly stable optimization trajectory that standard ResNets lack. Together with systematic checkpointing and intermediate feature monitoring, these measures ensure a robust, stable training process and enhance interpretability.

**Fig. 3:**
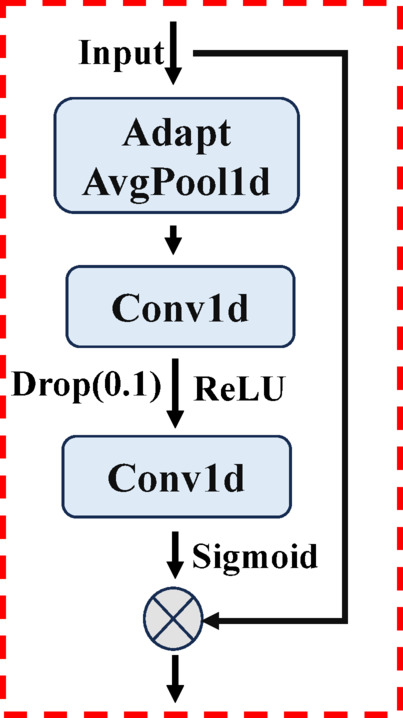
Channel attention mechanism. Global contextual information is first aggregated via adaptive average pooling, followed by two sequential Conv1D layers that capture inter-channel dependencies and generate attention weights. These weights are then multiplied by the input feature map to adaptively recalibrate channel-wise feature responses.

We further integrate channel attention into each residual unit as a lightweight, learnable reweighting mechanism over convolutional channels (interpretable as derived feature subspaces), the channel attention mechanism is shown in Fig. 3. The detailed formulations of the residual connection and channel-attention mechanism are provided in Eqs. (5) and (6), where $F(x, W_i)$ denotes the residual mapping with parameters $W_i$, $x$ is the input, and GAP stands for global average pooling.

5 $$y=F(x, W_i) + x$$

6 $$\hbox{Attention}(x)= \sigma (W_2\cdot \hbox{ReLU} (W_1\cdot \, \hbox{GAP}(x)))$$

Following the Squeeze-Excitation design, we apply global average pooling along the sequence dimension to obtain channel-wise statistics, pass them through a two-layer gating module with an intermediate nonlinearity and a final sigmoid to produce per-channel weights in the range (0, 1), and then scale the residual features channel by channel. As the weights vary, the block interpolates between an identity mapping through the skip connection (weights approaching 0) and a standard residual transformation (weights approaching 1), thereby preserving the stable optimization behavior of ResNets. After feature selection, although the input comprises only about six normalized clinical indicators, the attention mechanism operates on learned convolutional channels rather than on the raw features. This design allows the model to further learn channel-wise importance and dynamically reallocate feature contributions with minimal parameter overhead. The strategy enhances feature utilization and discriminative power, yields smoother validation accuracy, and improves robustness to mild distribution shifts and noise while maintaining unobstructed gradient flow.

### Statistical metrics

**Fig. 4:**
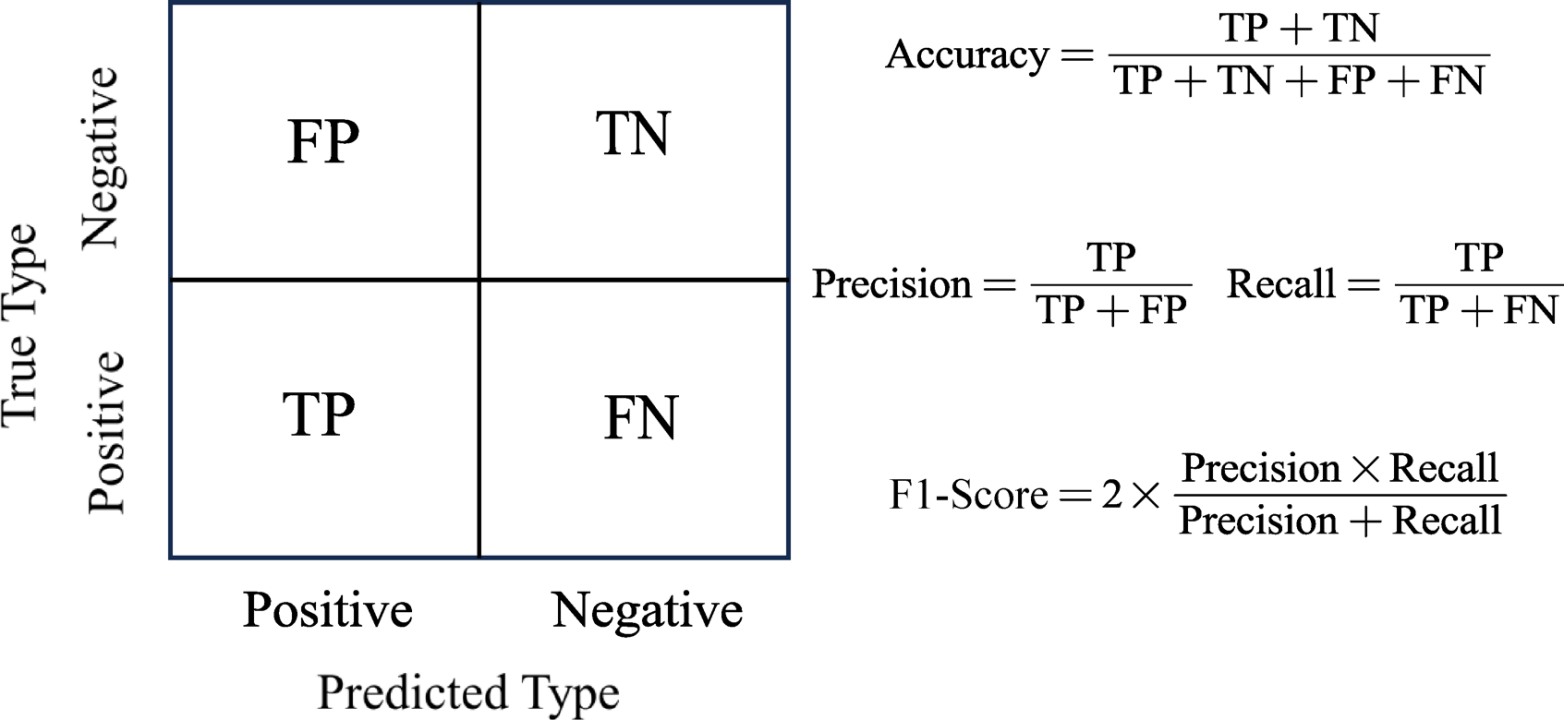
**Evaluation metrics.** Mathematical definitions and calculation formulas for accuracy, precision, recall, and F1-score based on TP, TN, FP, and FN.

To evaluate model performance, a suite of statistical metrics was employed^41,42^, including the confusion matrix, accuracy, precision, recall, F1-score, and the ROC curve. True positives (TP), true negatives (TN), false positives (FP), and false negatives (FN) denote the elements of the confusion matrix (Fig. 4). Accuracy provides an overall measure of the classifier’s correctness. Precision quantifies the proportion of predicted positive samples that are truly positive, reflecting the model’s ability to avoid false positives. Recall measures the proportion of actual positives correctly identified, reflecting the model’s ability to capture positive instances. The F1-score, the harmonic mean of precision and recall, summarizes the trade-off between these two metrics; a higher F1-score indicates more balanced and robust performance. Moreover, we trained ProgMDD with the standard multi‑class cross‑entropy loss and monitored both training and validation loss to characterize the optimization dynamics.

### Ablation study design

To rigorously evaluate the individual contribution of each architectural component within the proposed ProgMDD framework, we designed a comprehensive ablation study. The standard 1D-ResNet was established as the baseline model. From this baseline, we systematically isolated specific modules—namely, the Progressive Regularization (Prog. Reg.) strategy, the Channel Attention (CA) mechanism, and the EMA strategy. By comparing the performance of the full ProgMDD architecture against these degraded variants (e.g., models trained without CA, without Prog. Reg., or without EMA), we aimed to quantify their respective impacts on mitigating overfitting, refining channel-wise feature representation, and stabilizing the optimization trajectory.

### Model visualization and interpretation

Deep neural networks often function as opaque models that lack explicit, human-interpretable decision rules. In our study, ProgMDD retains a compact architecture after LASSO-based feature selection, yielding six key clinical variables (Gender, Age, HbA1c, Chol, TG, BMI). To examine how the network organizes samples, we extract the latent representation from the first fully connected layer and project it into two dimensions using UMAP (n_neighbors = 15, min_dist = 0.1, n_components = 2, random_state = 42), revealing clear inter-class separation that complements standard performance metrics. For feature attribution, we compute SHAP values on the held-out test set to obtain both class-wise and global (mean-absolute) importance, and cross-validate these attributions with the multi-class LASSO ($\lambda.1se$)-selected subset. The consistent ranking and direction of effects between SHAP and LASSO provide convergent evidence that these variables drive the model’s predictions, thereby enhancing credibility and clinical interpretability.

## Results

### Clinical parameter distributions

**Fig. 5:**
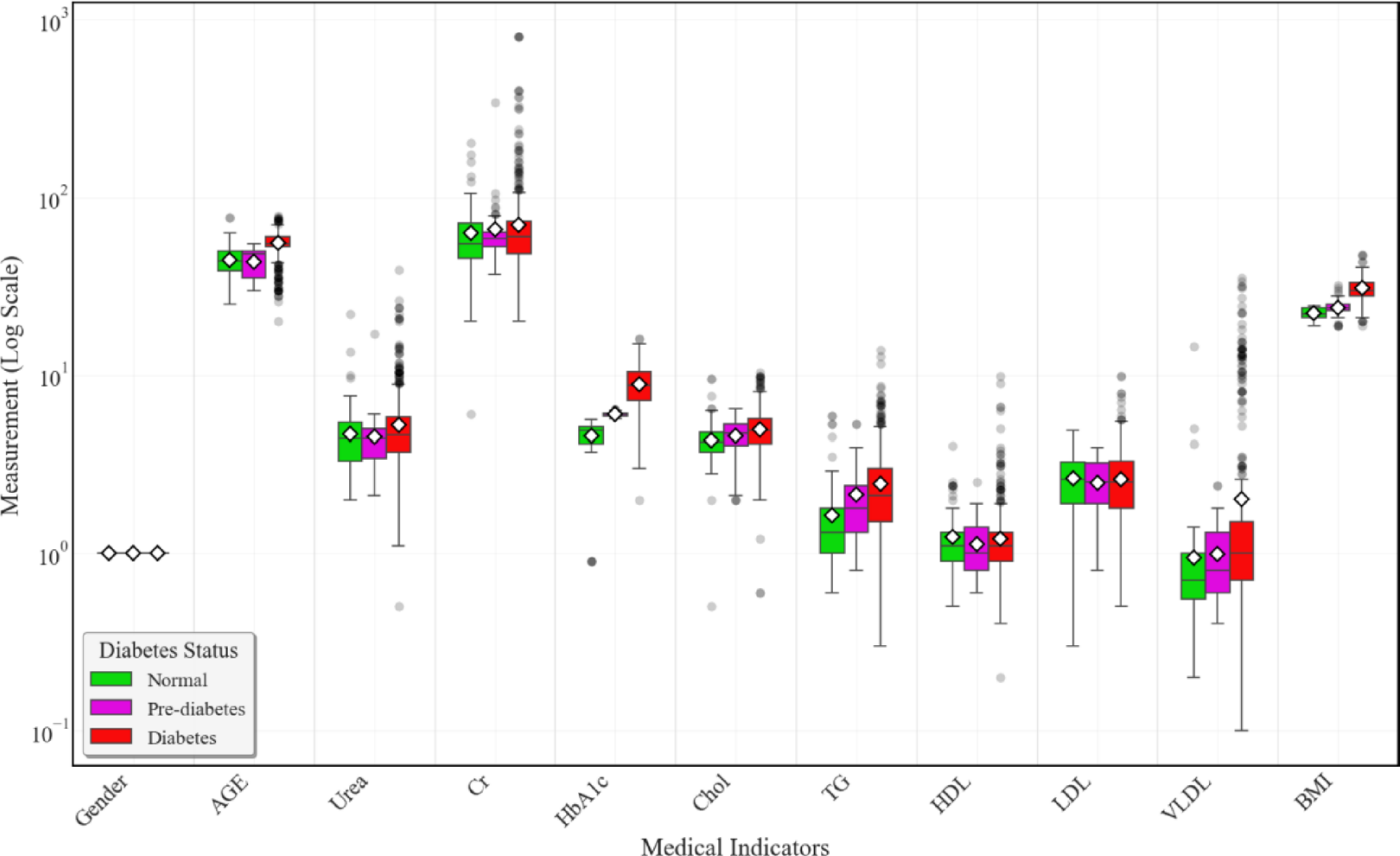
Box plots of eleven clinical biomarkers across Non-Diabetic, Pre-Diabetic, and Diabetic groups.

Figure 5 presents biomarker distributions across different diabetes status groups in the original dataset, revealing distinct patterns among Non-Diabetic (N), Pre-Diabetic (P), and Diabetic (Y) categories. The Diabetic group demonstrates significantly elevated levels in glucose metabolism markers (HbA1c) and renal function indicators (Cr, Urea). At the same time, lipid profiles (TG, LDL, VLDL) show pronounced dysregulation compared to the Non-Diabetic and Pre-Diabetic groups. Pre-Diabetic patients exhibit intermediate biomarker profiles, serving as a transitional state, whereas Non-Diabetic patients maintain optimal ranges across all measured parameters, particularly in metabolic homeostasis markers.

### Dimension reduction analysis

**Fig. 6:**
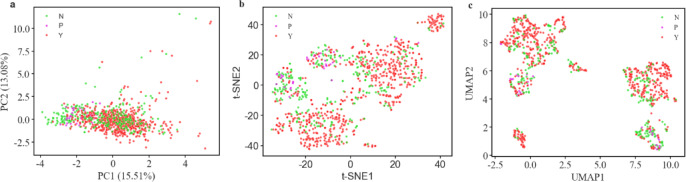
Dimensionality reduction of raw features. (**a**), (**b**), (**c**) representing two-dimensional embeddings using PCA, t‑SNE, and UMAP, respectively. (**a**) PCA explains little variance and shows only partial separation between Diabetic and Non-Diabetic, with Pre-Diabetic broadly overlapping both. (**b**) t‑SNE reveals locally compact neighborhoods for Diabetic and Non-Diabetic, while Pre-Diabetic remains dispersed among them; note that the global layout and inter-cluster distances are not interpretable. (**c**) UMAP yields tighter grouping and improved Non-Diabetic distinction compared with PCA and t‑SNE, yet Pre-Diabetic continues to interleave, indicating limited separability in unsupervised embeddings. All panels use standardized raw features without prior feature selection; class imbalance is present.

As illustrated in Fig. 6a, PCA explains 15.5% and 13.1% of the variance through its first two components, respectively. However, it fails to yield meaningful separation, with the three categories (N, P, Y) showing extensive overlap. This suggests that the discriminative structure of the clinical features is inherently nonlinear, particularly for the P group, which reflects the biological reality of prediabetes as a transitional state.

In comparison, t-SNE (Fig. 6b) improves the visualization of local neighborhood structures, but the resulting clusters remain fragmented and scattered. The P category continues to overlap with both N and Y, indicating that while t-SNE captures fine-grained substructures, it lacks reliable global arrangement and distinct class boundaries.

UMAP (Fig. 6c) demonstrates the most cohesive clustering by preserving global topology while maintaining local neighborhoods. Although it reduces fragmentation and yields more compact groupings compared to PCA and t-SNE, significant overlap remains among the categories. This reinforcement of blurred boundaries across the health-to-disease spectrum underscores the complexity of identifying the pre-diabetic stage.

Collectively, these visualization methods consistently reveal substantial inter-class overlap and the challenges of distinguishing transitional states in unsupervised, low-dimensional embeddings. Clinically, these obscured boundaries reflect a continuum of disease progression rather than discrete partitions, emphasizing the necessity for robust supervised models and effective data preprocessing to extract discriminative signals for early detection.

### Data processing and feature selection

**Fig. 7:**
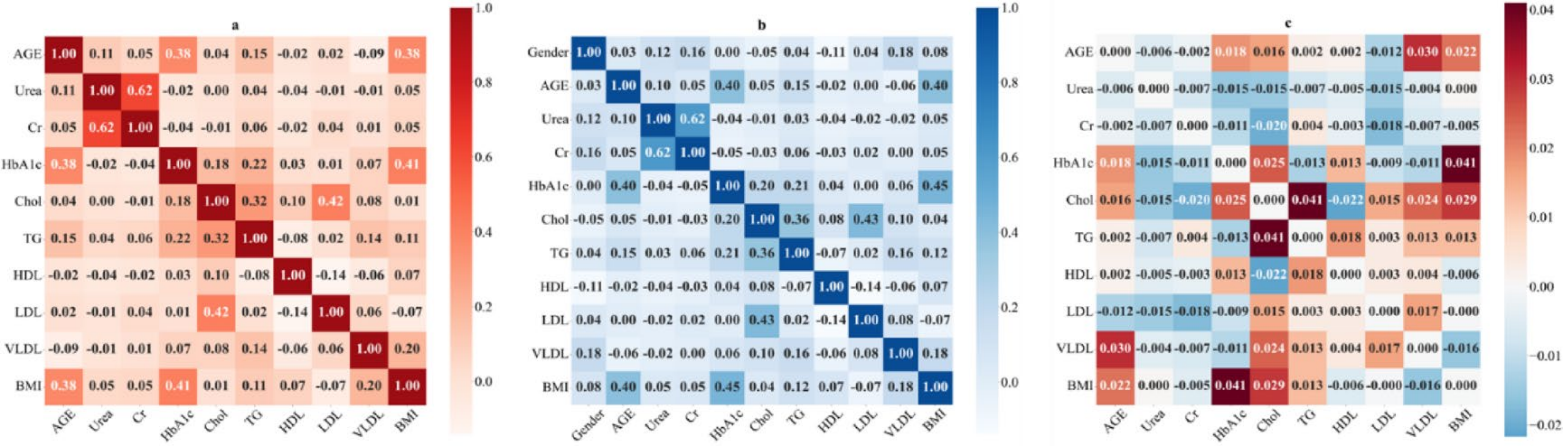
Correlation analysis. Pearson correlation heatmaps for (a) original and (b) processed datasets, with (c) the difference matrix (processed − original).

To verify that the synthetic samples generated via SMOTE-Tomek remain consistent with clinical reality, we analyzed the Pearson correlation structures before and after preprocessing. Figure 7 presents the Pearson correlation heatmaps for the original dataset (panel a) and the preprocessed dataset (panel b), together with the element-wise difference matrix (processed−original, panel c). Importantly, these preprocessing and balancing steps were implemented strictly within the training partition to prevent data leakage. Rather than assessing feature importance, which is systematically addressed later via LASSO regression, this analysis specifically focuses on the internal feature-to-feature covariance. In this context, Pearson correlation is employed purely as a descriptive measure to characterize pairwise relationships among continuous variables, without implying causality or statistical inference. Several canonical biological relationships are evident in the original dataset, including AGE-HbA1c (*r* = 0.38), Urea-Cr (*r* = 0.62), HbA1c-BMI (*r* = 0.41), Chol-TG (*r* = 0.32), and Chol-LDL (*r* = 0.42). After SMOTE-Tomek preprocessing, these global patterns remain remarkably stable. Feature-feature dependencies are preserved with only minor acceptable shifts (e.g., HbA1c-BMI increasing slightly to 0.45, Chol-TG to 0.36). The difference matrix (panel c) confirms that the absolute changes (Δr) for the vast majority of feature pairs remain near zero. These findings collectively suggest that the resampling step successfully preserves the global biological correlation structure without introducing synthetic artifacts, ensuring that the model learns from authentic clinical patterns. To ensure complete transparency in our experimental setup, the exact distribution of original and synthetic samples across the data splits is summarized in Table 2.

**Table 2:** **Sample accounting across experimental splits.** Resampling is used only to balance the training set. Validation and testing are performed entirely on original clinical data. Y denotes Diabetic, N denotes Non-Diabetic, and P denotes Pre-Diabetic.

Dataset Split	Class	Original	Synthetic	Total
Training (70%)	Y	486	0	486
	N	67	419	486
	P	28	458	486
Validation (10%)	Y	69	0	69
	N	10	0	10
	P	4	0	4
Testing (20%)	Y	139	0	139
	P	19	0	19
	N	8	0	8
Total		830	877	1707

**Fig. 8:**
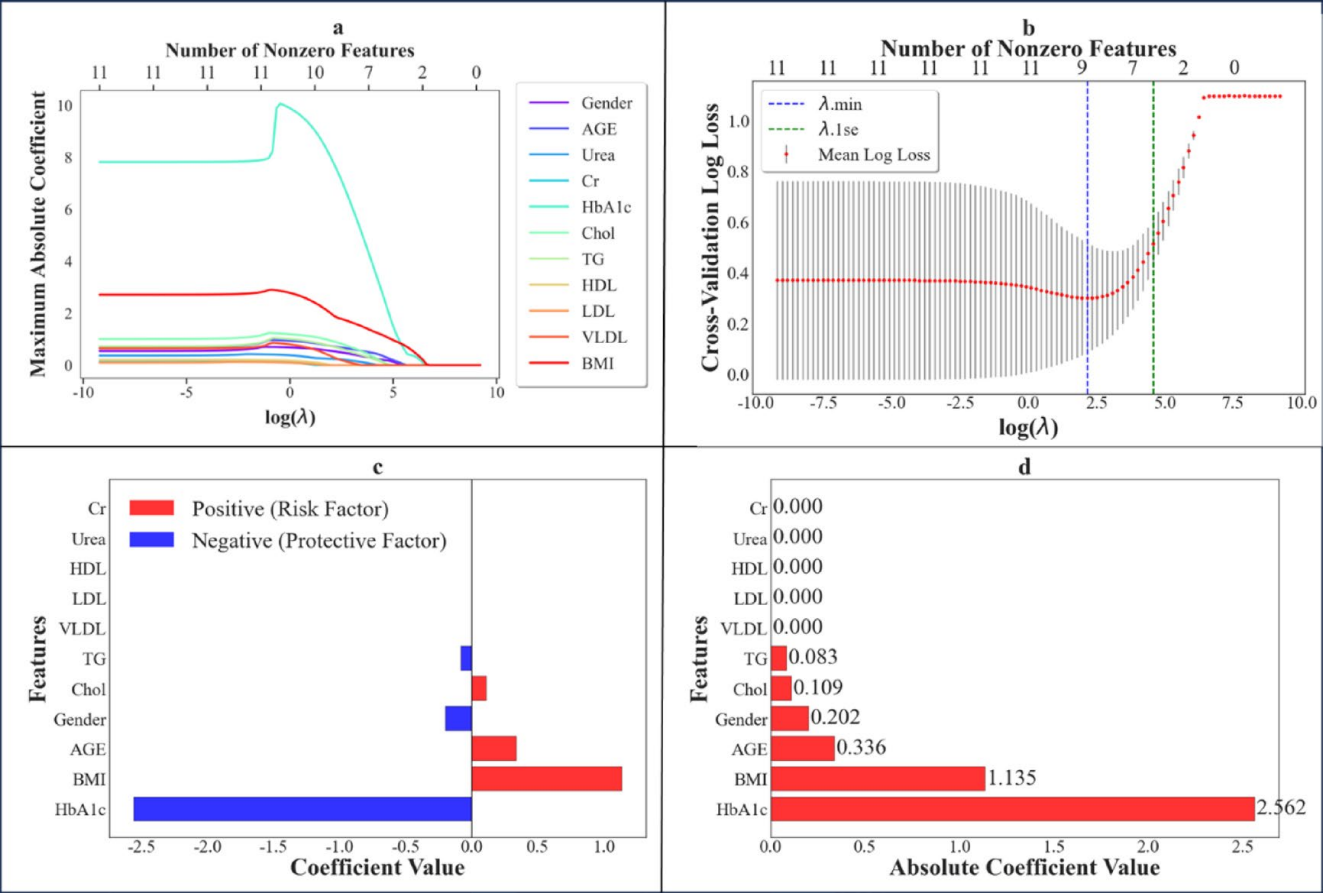
**LASSO feature selection.** (a) Coefficient paths across 100 logarithmically spaced regularization levels (*log*(*λ*) from − 4 to + 4). (b) 10-fold cross-validation log-loss curve with standard error bars; *λ_min* = 8.498 (8 non-zero features) and *λ_1se* = 95.455 (6 non-zero features). (c) Directional feature importance at *λ_1se* with signed coefficients (red: positive; blue: negative). (d) Absolute feature importance at *λ_1se*, ranking predictors by coefficient magnitude.

Following the data partitioning, we applied LASSO to the standardized training set, before data augmentation, to identify the most robust clinical predictors and further mitigate the risk of data leakage. We evaluated the feature selection process across 100 logarithmically spaced regularization levels (Fig. 8a; $log(\lambda) \in [ - 4, + 4]$) using 10‑fold cross‑validation (Fig. 8b). The minimum cross‑validated log‑loss occurred at $\lambda_{min} = 8.498$, yielding eight non‑zero features (Gender, AGE, Urea, HbA1c, Chol, TG, VLDL, BMI). Meanwhile, the 1‑SE solution at $\lambda_{1se} = 95.455$ yielded a sparser six‑feature model (Gender, AGE, HbA1c, Chol, TG, BMI). Coefficient paths show that high‑signal features (HbA1c, BMI, AGE) remain non‑zero across wide regularization ranges, whereas weaker signals (e.g., Urea, VLDL) shrink to zero under stronger penalization. Directional importance at $\lambda_1se$ (Fig. 8c) reports signed representative coefficients per feature, and absolute importance (Fig. 8d) ranks features by $|coef |$. Notably, HbA1c and BMI dominate the selection, while AGE, TG, and Chol provide secondary contributions. These patterns are highly consistent with the correlation structure observed in Fig. 7. To favor model parsimony and ensure optimal generalization, we adopted the $\lambda_{1se}$ feature set for subsequent model training.

### ProgMDD prediction and visualization

Feature selection was applied to the training set only after the initial partition. This protocol ensures that the validation and test sets remain entirely representative of the real-world clinical distribution. It is worth noting that this analysis is based on the experimental procedures and results conducted by ProgMDD on the original, unbalanced dataset. The model was fitted on the unbalanced training data, while the validation set was utilized for monitoring convergence and hyperparameter tuning.

**Fig. 9:**
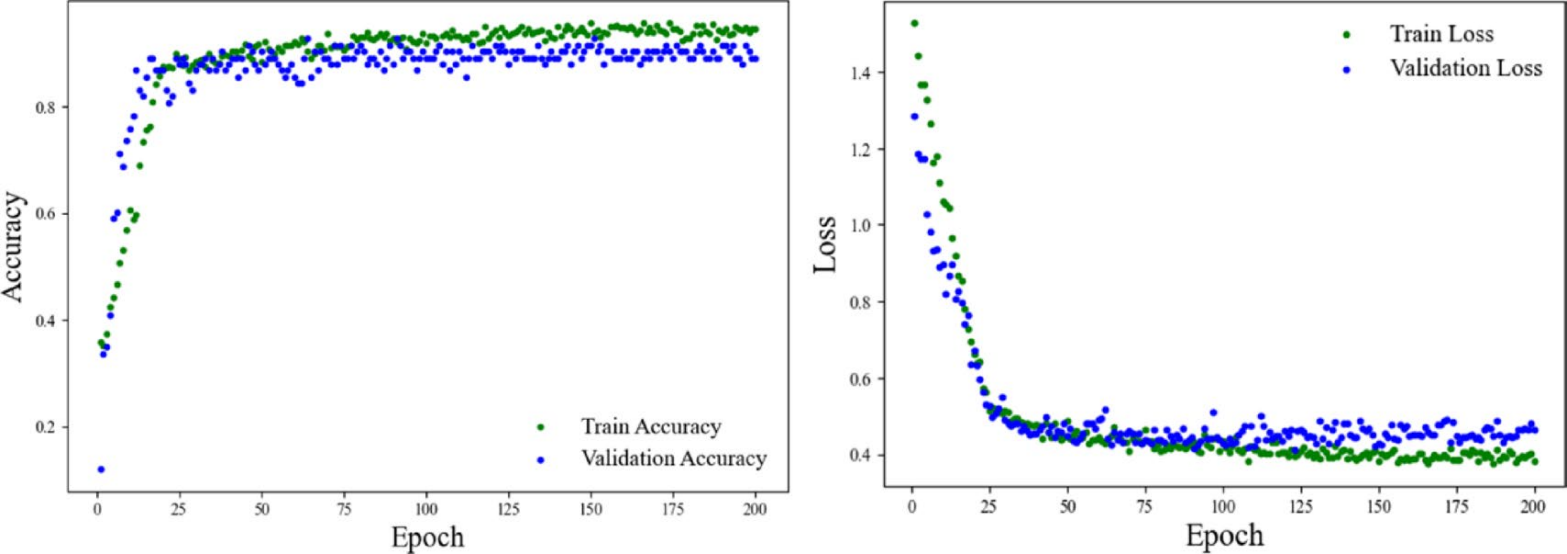
**Training dynamics.** The horizontal axis represents epochs, indicating training iterations. As the number of epochs increases, the model’s loss and accuracy gradually converge.

Figure 9 illustrates the evolution of loss and accuracy across 200 training epochs. At epoch 1, the model was in an underfit state with accuracies of 35.80% for training and 22.05% for validation, which is expected following random weight initialization. As training proceeded, the loss decreased and accuracy increased rapidly. By epoch 40, performance reached 89.50% in training and 86.59% in validation. During the final stage of training, we observed minor stochastic fluctuations in the validation loss. This is a common phenomenon in clinical studies with limited data sizes, but the overall trend confirms a robust convergence. By epoch 150, the model already exhibited reasonable discriminative capabilities, with training and validation accuracies reaching 95.70% and 91.16%, respectively. The performance curves gradually plateaued at a high level, demonstrating that progressive regularization effectively alleviated early overfitting.

**Fig. 10:**
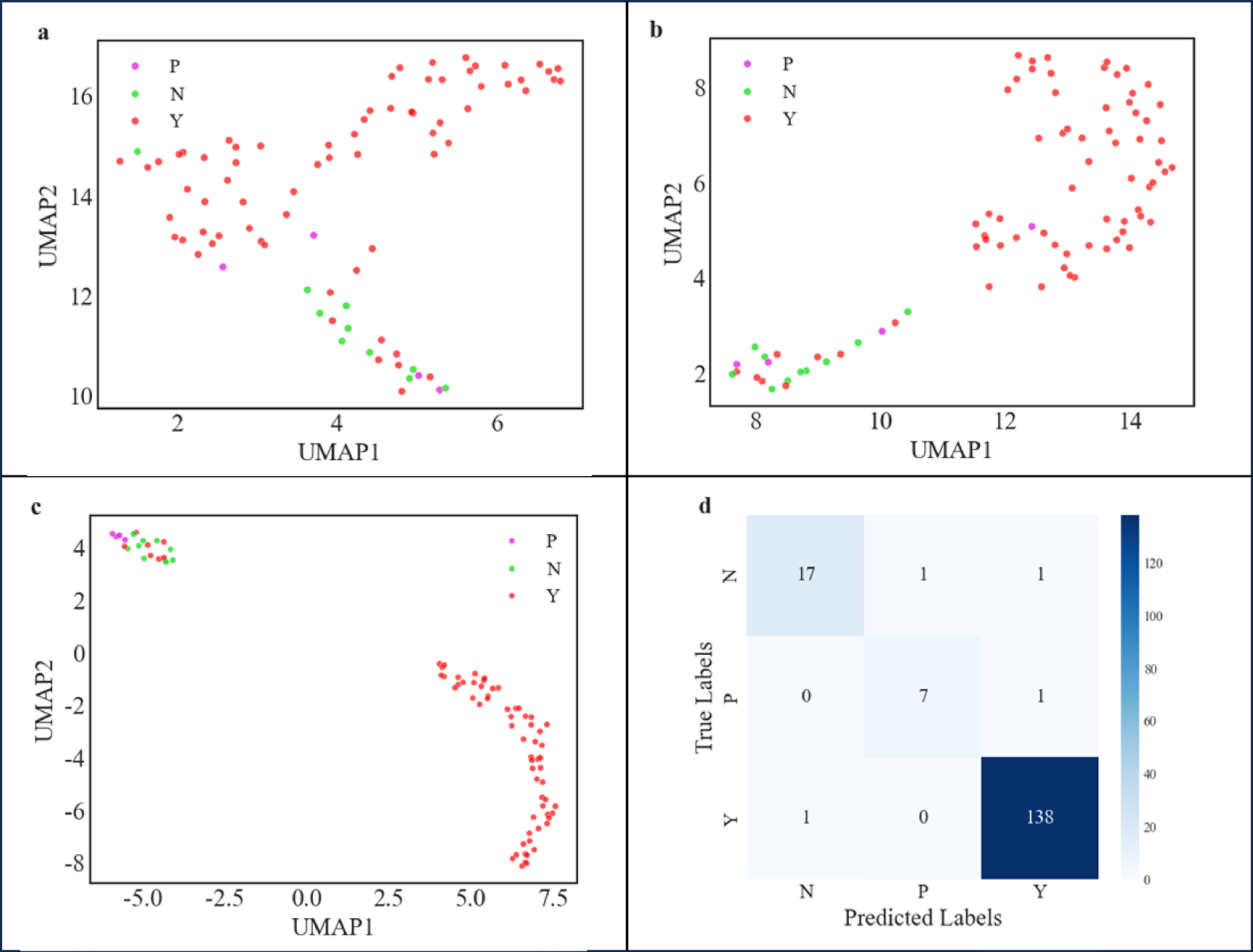
**Progressive feature learning and classification.** (a), (b), (c) correspond to the visualizations of the 1st, 40th, and 150th epoch, respectively. (d) is the confusion matrix representing the prediction accuracy of the test set in the 150th epoch model. From (a), (b), and (c), it can be observed that with increasing training epochs, the discrimination accuracy improves, and the scatter points of the same type of diabetes become more clustered.

To examine the learned feature representations, we applied UMAP to the activations of the first fully connected layer (relu_fc1) and visualized the validation set at epochs 1, 40, and 150. As shown in Fig. 10a–c, the three diabetes classes become progressively more distinct as the number of epochs increases. At epoch 1, the features are randomly distributed and show no initial class structure. By epoch 40, clustering begins to emerge, although significant overlap remains between all categories. By epoch 150, the classes are markedly more distinct. While some subtle overlap persists between the Pre-Diabetic and Non-Diabetic clusters because of the high clinical and biological similarity between these adjacent physiological stages, the Diabetic class is almost entirely isolated from the others.

The confusion matrix on the held-out test set using the optimal validation checkpoint confirms these findings (Fig. 10d). The matrix exhibits a dominant diagonal structure, indicating high precision across all classes. Consistent with the UMAP visualization, residual misclassifications are primarily concentrated between the Pre-Diabetic and Non-Diabetic categories. This underscores the inherent challenge of early-stage diabetes stratification in real-world clinical settings where biological signals often overlap.

### Model estimation

To evaluate the predictive capability of ProgMDD under real-world clinical conditions, we first assessed its performance on the completely unaugmented, held-out test set (Table 3). This initial evaluation establishes a baseline on the original imbalanced data distribution without the intervention of resampling techniques. When this raw test set (*n* = 166) was fed into the trained model, it achieved a strong overall accuracy of 97.59%. Given the severe natural class imbalance, the majority Diabetic class (support = 139) dominated the dataset and achieved near-perfect scores, with precision, recall, and F1-score all reaching 0.99. Importantly, the model also successfully identified the extreme minority groups. The Non-Diabetic class (support = 19) attained a precision of 0.94, a recall of 0.89, and an F1-score of 0.92, while the Pre-Diabetic class (support = 8) achieved a precision of 0.88, a recall of 0.88, and an F1-score of 0.88. The macro-average F1-score reached 0.93, indicating that the model maintains a highly competitive predictive capacity and learns meaningful features even without data balancing.

**Fig. 11:**
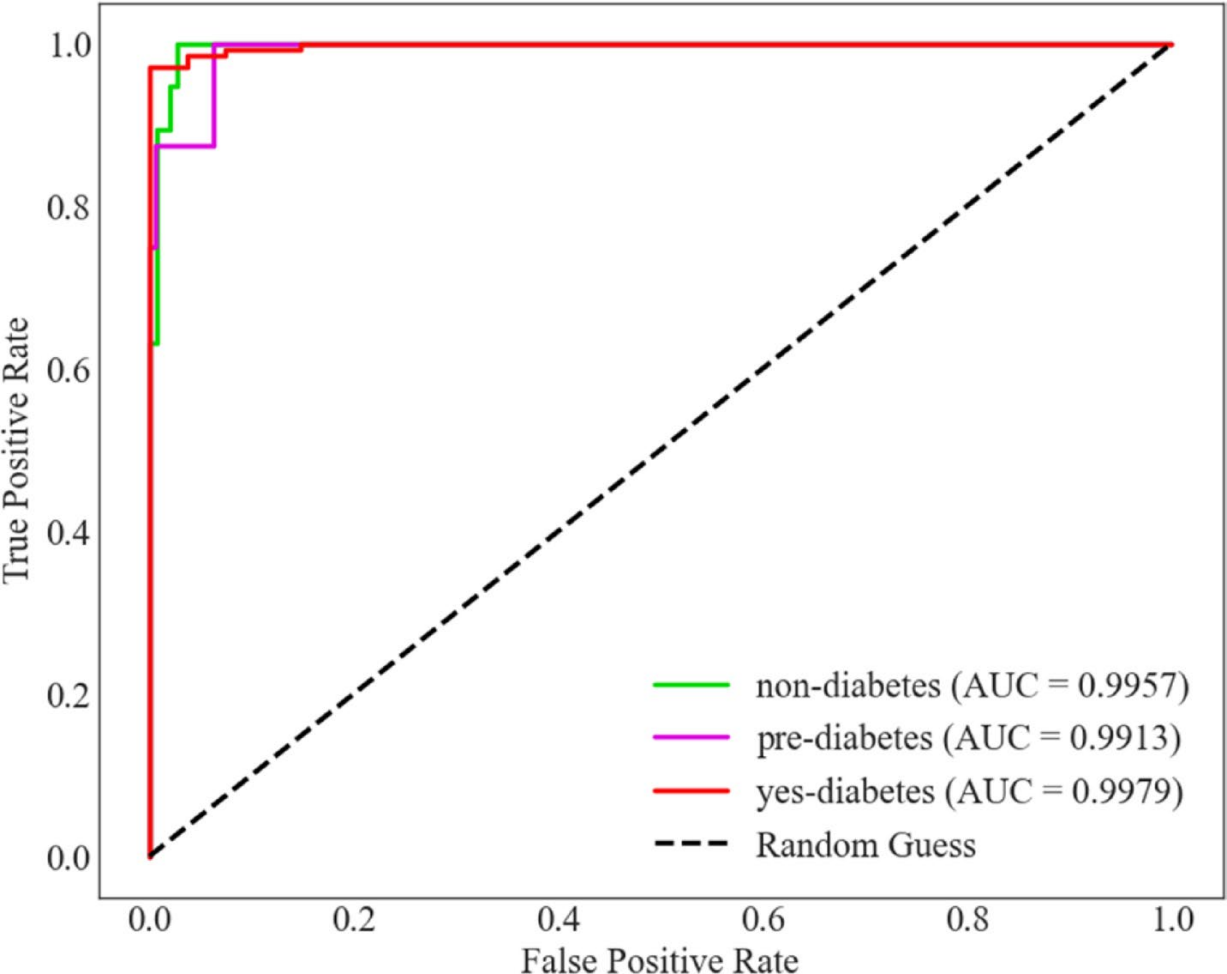
ROC analysis. Receiver Operating Characteristic curves and AUC values for multiclass diabetes diagnosis.

This discriminative performance is further supported by the ROC curve analysis for this baseline evaluation (Fig. 11). The area under the ROC curve (AUC) values were highly robust across all categories: 0.9957 for the Non-Diabetic class, 0.9913 for the Pre-Diabetic class, and 0.9979 for the Diabetic class. The resulting macro-average AUC of 0.9960 confirms that the architecture can confidently separate clinically adjacent categories despite the significant scarcity of minority training samples.

While the baseline results on the imbalanced test set are highly promising, our primary validation relies on a rigorous stratified 5-fold cross-validation. This experiment is the most critical evaluation of our study because it fully integrates our balancing strategy while strictly adhering to the nested protocol described in the Method. To explicitly address sample bias and completely avoid data leakage, the SMOTE-Tomek resampling was applied exclusively to the training folds during each iteration. This allowed the model to better learn the minority-class decision boundaries, while the validation folds and the test sets were left entirely in their original, unaugmented state for unbiased testing.

Under this strict and comprehensive evaluation framework, ProgMDD demonstrated highly consistent and stable performance. As summarized in Table 4, the model yielded a mean accuracy of 97.02% (± 1.11%) and a mean precision of 0.970 (± 0.006) across 5-fold cross-validation., with a narrow 95% Confidence Interval for accuracy between 95.64% and 98.40%. This low variance across different data partitions substantiates the model’s robustness. These findings collectively confirm that the architecture effectively captures authentic biological patterns rather than overfitting to synthetic artifacts. Overall, this combination of strong baseline capability and statistically stable cross-validation provides rigorous evidence that ProgMDD is a reliable and generalizable tool for multiclass diabetes stratification in clinical settings. To ensure transparency and reproducibility, comprehensive details regarding the exact experimental settings, hardware platforms, and the model’s computational complexity are provided in Supplementary Note 1.

**Table 3:** Model performance on the original imbalanced dataset.

Metrics	Precision	Recall	F1-score	Support
Non-Diabetic	0.94	0.89	0.92	19
Pre-Diabetic	0.88	0.88	0.88	8
Diabetic	0.99	0.99	0.99	139
**Overall accuracy**			0.9759	166

**Table 4:** Performance of ProgMDD under stratified 5-fold cross-validation.

Fold	Accuracy (%)	Precision	Recall
Fold 1	97.59%	0.976	0.976
Fold 2	96.78%	0.963	0.958
Fold 3	96.99%	0.971	0.980
Fold 4	96.37%	0.975	0.974
Fold 5	98.39%	0.975	0.986
Mean (± *SD*)	97.02 ± 1.11	0.970 ± 0.006	0.975 ± 0.010
95% CI	[95.64, 98.40]	[0.963, 0.977]	[0.962, 0.988]

### Comparative evaluation with baseline models

To comprehensively evaluate the incremental value of ProgMDD, we compared it against six well-established baseline models. To ensure absolute fairness, all models were trained and evaluated under the exact same rigorous protocol. We used stratified 5-fold cross-validation, and crucially, SMOTE-Tomek resampling was applied strictly within the training folds. The validation folds retained their original, imbalanced data distribution to reflect real-world clinical scenarios. The comparative results are summarized in Table 5.

Clinical Rule-based and Basic Deep Learning Baselines. We first established a “Rule-based Baseline” using a Decision Tree limited to a maximum depth of 3. We intentionally restricted this model to use only three dominant variables: HbA1c, Age, and BMI. This design simulates a standard, transparent clinical heuristic used by human doctors. This basic rule achieved a strong accuracy of 95.65%. In contrast, a Basic MLP achieved 95.10%. This indicates that simply applying standard deep learning without architectural innovation fails to outperform basic clinical rules on this specific tabular dataset.

Traditional and State-of-the-Art (SOTA) Machine Learning. We also tested traditional and advanced machine learning models. Multinomial Logistic Regression and SVM achieved accuracies of 90.84% and 93.13%, respectively. We then evaluated Random Forest and XGBoost, which are widely considered SOTA for tabular data tasks. Both ensemble models performed strongly, with XGBoost reaching an impressive accuracy of 96.47% and a precision of 0.971.

Superiority of ProgMDD. Despite the strong performance of XGBoost, our proposed ProgMDD model achieved the best overall results with an average accuracy of 97.02%. More importantly, ProgMDD achieved the highest recall score of 0.975. In medical diagnostics, a high recall is particularly critical because it minimizes false negatives and ensures that potential patients are not missed.

Overall, these comparisons clearly demonstrate the necessity of our model design. While simpler models and clinical rules provide a solid baseline based on a few key biomarkers, the progressive residual architecture of ProgMDD successfully captures deeper, complex physiological patterns to provide the most reliable and sensitive diagnostic performance. Notably, standard machine learning baselines evaluated in our study, such as Random Forest, XGBoost, and SVM, are inherently order-agnostic tabular architectures. The fact that ProgMDD maintains a significant performance advantage over these models indicates that its superior predictive capability stems from deep hierarchical feature interactions and robust regularization, rather than being an artifact of specific variable ordering.

**Table 5:** Performance comparison of ProgMDD against baseline models. All models were evaluated using a stratified 5-fold cross-validation protocol. Resampling was strictly confined to the training folds. The best results for each metric are highlighted in bold.

Model	Accuracy (%)	Precision	Recall
Multinomial LR L1	90.84 ± 2.19	0.943 ± 0.009	0.908 ± 0.022
SVM	93.13 ± 1.93	0.943 ± 0.013	0.931 ± 0.019
Random Forest	96.17 ± 0.9	0.962 ± 0.010	0.961 ± 0.011
XGBoost	96.47 ± 0.8	**0.971 ± 0.005**	0.968 ± 0.005
Basic MLP	95.10 ± 1.67	0.956 ± 0.017	0.956 ± 0.017
Rule-based Baseline (Decision Tree)	95.65 ± 1.93	0.964 ± 0.014	0.961 ± 0.019
ProgMDD (Ours)	**97.02 ± 1.11**	0.970 ± 0.006	**0.975 ± 0.010**

### Ablation and sensitivity analysis

To quantify the individual contribution of each core component, we conducted a comprehensive ablation study using the original, unaugmented dataset. The quantitative results of the ablation study, including precision, recall, F1-score, and overall accuracy, are summarized in Table 6.

Performance of the Baseline. The baseline 1D-ResNet achieved an accuracy of 93.77% and an F1-score of 0.93. While residual connections provide a foundation for feature extraction, this lower performance shows that standard convolutions alone cannot fully capture the complex physiological patterns of early-stage diabetes.

Contribution of Progressive Regularization. Removing Progressive Regularization (w/o Progressive Reg.) caused the accuracy to drop to 95.30%. This decrease indicates that dynamically adjusting regularization intensity is necessary to balance bias and variance. Without this mechanism, the model is more prone to overfitting during the early stages of training, which destabilizes the optimization process in high-noise and small-scale feature spaces.

Impact of Channel Attention. The removal of the Channel Attention module (w/o Channel Attention) resulted in a significant performance decline, with accuracy falling to 95.78% and the F1-score to 0.95. This confirms that the attention mechanism is a primary driver of model performance. It recalibrates feature responses by suppressing noise from irrelevant indicators and focusing the network on the most informative biomarkers. Its absence leads to a dilution of critical biological signals, making it difficult for the model to isolate minority-class characteristics from background noise.

Efficacy of the EMA Strategy. Excluding the EMA strategy (w/o EMA Strategy) led to an accuracy of 96.99% and an F1-score of 0.97. Although the performance gap is smaller than other modules, the EMA strategy serves as a vital temporal ensemble technique. It smooths the decision boundaries and prevents the model from converging to unstable local optima. In clinical settings, this stability is crucial for ensuring consistent and reliable diagnostic results across different patient profiles.

Overall Synergy. The full ProgMDD model integrates all these modules to achieve an optimal accuracy of 97.59% and an F1-score of 0.98. This 3.82% improvement over the baseline proves that these components are highly synergistic. Progressive Regularization ensures stable convergence, Channel Attention refines the feature representations, and the EMA strategy secures robust generalization.

**Table 6:** Ablation study results. “w/o” denotes the removal of a specific component from the full ProgMDD framework. The best results are highlighted in bold.

Model Variant	Precision	Recall	F1-score	Overall accuracy (%)
Baseline (1D-ResNet)	0.94	0.93	0.93	93.77%
w/o Progressive Reg.	0.95	0.95	0.96	95.30%
w/o Channel Attention	0.96	0.96	0.95	95.78%
w/o EMA Strategy	0.97	0.97	0.97	96.99%
**ProgMDD (Full)**	**0.98**	**0.97**	**0.98**	**97.59%**

In addition to component ablation, we further evaluated the model’s sensitivity to input feature structures. As clinical tabular data (comprising purely discrete variables such as AGE, BMI, and HbA1c) lacks natural spatial adjacency, a standard 1D-CNN might theoretically impose artificial local structures depending on the chosen feature order. To verify that ProgMDD’s performance gain is not merely an artifact of variable ordering, we conducted a feature permutation sensitivity analysis.

The input order of the six selected biomarkers was randomly shuffled into five distinct sequential configurations, and the ProgMDD model was retrained under identical hyperparameter settings for each configuration. The results are summarized in Table 7. ProgMDD demonstrated remarkable stability across all random permutations, yielding a mean accuracy of 97.12% (± 0.58%) and a mean F1-score of 97.03% (± 0.63%). The minimal variance in predictive performance empirically proves that the architecture is robust to spatial adjacency perturbations. This stability is primarily attributed to the Channel Attention mechanism, which utilizes Global Average Pooling (GAP) to assess the global context and dynamically recalibrate feature importance independently of their sequential positions.

**Table 7:** Sensitivity analysis of ProgMDD under random feature permutations. The feature sequential order for each configuration is defined as follows: Exp-1 (Gender, AGE, HbA1c, Chol, TG, BMI); Exp-2 (BMI, TG, Chol, HbA1c, AGE, Gender); Exp-3 (AGE, Gender, BMI, HbA1c, TG, Chol); Exp-4 (HbA1c, Chol, TG, BMI, Gender, AGE); Exp-5 (TG, BMI, AGE, Gender, Chol, HbA1c).

Configuration	Precision	F1-score	Accuracy(%)
Experiment-1	0.9704	0.9718	97.28
Experiment-2	0.9629	0.9616	96.39
Experiment-3	0.9677	0.9660	96.58
Experiment-4	0.9790	0.9767	97.59
Experiment-5	0.9805	0.9752	97.78
Mean (± *SD*)	0.9721(± 0.0076)	0.9703(± 0.0063)	97.12(± 0.58)

### SHAP values

**Fig. 12:**
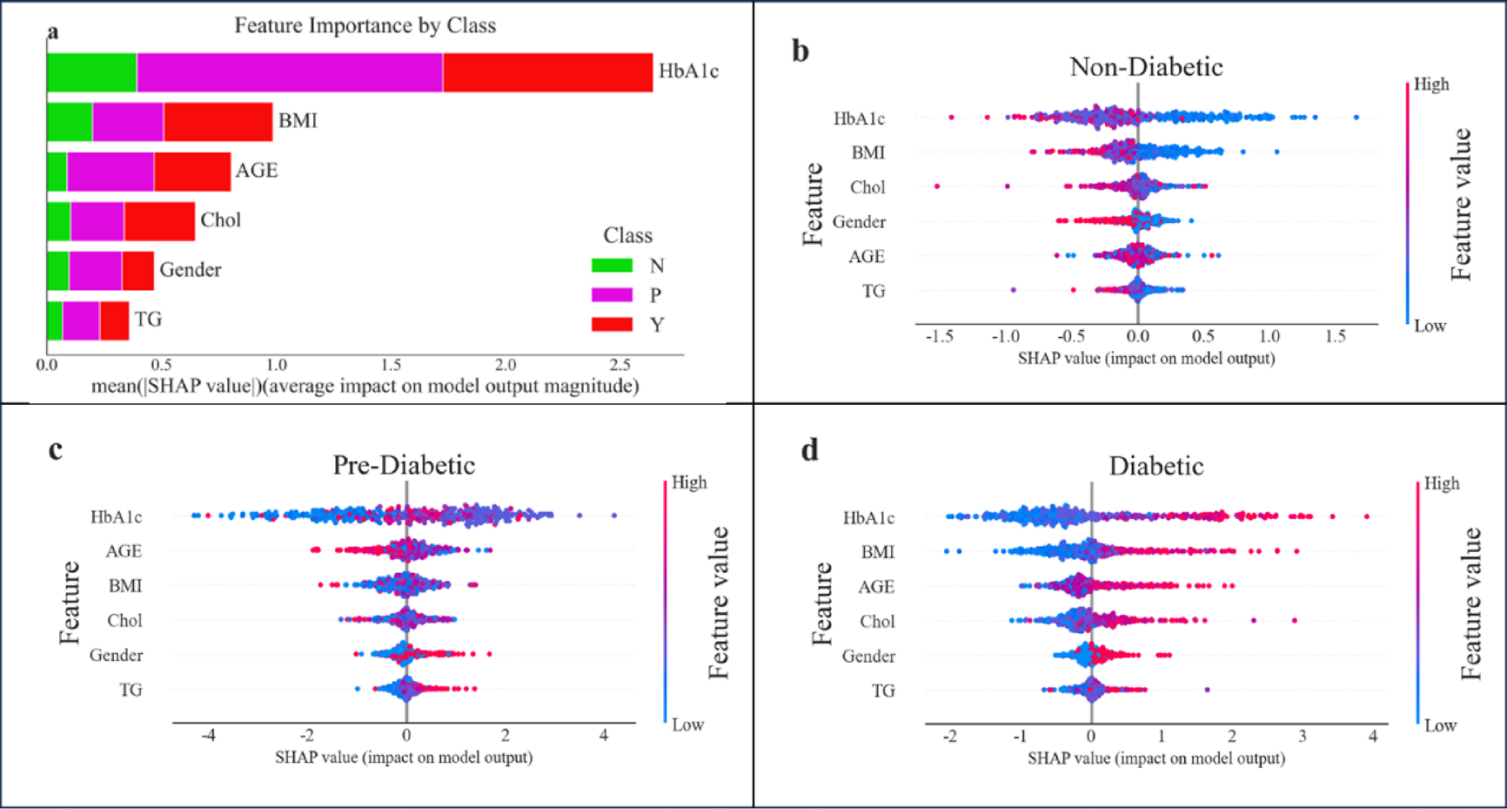
SHAP interpretability analysis. (a) Mean absolute SHAP values illustrating the global feature importance for three types of diabetes diagnosis. The horizontal axis denotes the mean absolute SHAP value, and the vertical axis lists six biomedical features ranked by importance. (b–d) Beeswarm plots visualize the feature-level SHAP distributions, highlighting each feature’s contribution to predicting specific diabetes categories.

As shown in Fig. 12a, the mean absolute SHAP values identify HbA1c, BMI, and AGE as the three most influential predictors, followed by Cholesterol, Gender, and TG. The top-ranked features collectively account for the majority of the model’s explanatory SHAP mass, while lower-ranked variables contribute progressively less to the decision process.

For the Non-Diabetic class (Fig. 12b), the SHAP distribution is dominated by low HbA1c and low BMI, both yielding the strongest positive impacts on Non-Diabetic predictions. Cholesterol ranks third, exerting a moderate but consistent influence, whereas AGE, Gender, and TG contribute marginally. In the Pre-Diabetic class (Fig. 12c), HbA1c, AGE, and BMI again emerge as the leading determinants. Moderate-to-high HbA1c, increasing AGE, and elevated BMI collectively drive the model toward Pre-Diabetic outcomes. Cholesterol and TG play secondary roles, and male gender shows a weak positive association in a subset of cases. For the Diabetic class (Fig. 12d), high HbA1c and high BMI remain the most decisive features, with AGE reinforcing the pattern observed in earlier stages. Cholesterol and Gender exhibit modest positive effects, while TG consistently represents the least influential factor across all categories.

In summary, HbA1c, BMI, and AGE dominate SHAP-based importance rankings throughout the clinical spectrum. This hierarchy aligns closely with the LASSO absolute coefficient analysis (Fig. 8d), which identifies the same leading predictors, despite slight variations in their relative magnitudes. The combined SHAP-LASSO evidence provides forward-backward validation of feature relevance, reinforcing the robustness and clinical interpretability of the model’s attributions.

## Discussion

The theoretical rationale for the selection of visualization algorithms is provided in Supplementary Note 2. In this study, the proposed ProgMDD achieved a robust overall accuracy of 97.02% under rigorous 5-fold cross-validation and 97.59% on an independent, unaugmented test set. This performance highlights the necessity of our specific architectural design. Rather than mechanically assembling deep learning components, ProgMDD was explicitly tailored for small-scale, high-noise tabular clinical data. By integrating progressive regularization with channel attention, the network dynamically increases penalty terms in deeper layers. This design effectively mitigates the severe overfitting risks typically associated with deep networks on small biomedical datasets, forcing the model to learn stable biological patterns rather than memorizing synthetic noise.

A key advantage of this framework lies in its alignment with established clinical guidelines. Feature attributions derived from SHAP reveal that HbA1c, BMI, and Age are the dominant predictors, which is directly consistent with American Diabetes Association (ADA) diagnostic standards. Our SHAP analysis provides a clear link between feature attributions and clinical decision thresholds. For instance, the model’s risk probability for the diabetic class increases sharply as HbA1c levels approach the 6.5% clinical threshold. This dual interpretability mechanism reinforces clinician confidence by triangulating predictive signals from both linear (LASSO) and non-linear (SHAP) perspectives.

Regarding clinical usability, ProgMDD is highly suitable for real-world deployment in primary care settings and community health-check centers. Because the model relies solely on routinely available biomarkers and basic anthropometric data, it minimizes operational burden and requires no specialized instrumentation. In clinical practice, ProgMDD is designed to function as an intelligent screening and decision-support tool rather than a standalone replacement for doctors. It is valuable for evaluating individuals whose metabolic profiles fall within the ambiguous transitional range (Pre-Diabetic), where routine limited panels often lead to experience-dependent interpretations. By providing transparent risk probabilities that align with actionable interventions like weight management, it bridges the gap between routine laboratory data and evidence-based early intervention.

Despite these strengths, several limitations must be acknowledged. First, the relatively small size of our cohort carries inherent challenges for multi-class learning; as observed in our results, residual errors are primarily concentrated in the minority classes, reflecting the natural difficulty of small-sample classification. Second, we acknowledge the lack of external multi-class validation. This is a common challenge due to the severe scarcity of public datasets containing a verified “Pre-diabetic” category. However, the Iraqi dataset utilized here is a recognized benchmark for Middle Eastern metabolic profiles and has supported multiple peer-reviewed studies. Future multi-center studies involving diverse populations and varying laboratory analyzers remain essential to verify calibration and ensure broad generalizability before guideline-level clinical translation.

## Conclusion

In this study, we proposed a progressive one-dimensional residual network with channel attention named ProgMDD for discriminating three types of diabetes based on biomarker sequences. Among three visualization methods (PCA, t-SNE, and UMAP), the UMAP algorithm was adopted for its ability to concurrently balance global and local perspectives in visualizing the model’s internal representations. To ensure methodological rigor and prevent data leakage, the original clinical records underwent a strict, leakage-free pipeline, where LASSO-based feature selection and SMOTE-Tomek resampling were implemented exclusively within the training partitions. This approach identified six key biomarkers as inputs, effectively enhancing model robustness while maintaining the integrity of evaluation.

The model’s discriminative capacity was rigorously validated on the original, imbalanced hold-out test set and through stratified 5-fold cross-validation, consistently achieving high diagnostic accuracy. Comprehensive benchmarking demonstrated that ProgMDD outperforms a diverse suite of baselines. Furthermore, ablation studies and feature permutation sensitivity analyses confirmed that the architectural advantages of ProgMDD stem from its inherent feature-learning capabilities rather than arbitrary variable ordering.

To address the intrinsic black-box nature of deep learning, SHAP analysis was conducted to quantify feature importance and cross-validate it against LASSO outcomes, confirming the stability and consistency of biomarker relevance. The findings underscore the critical roles of HbA1c, body mass index (BMI), and other biomarkers in differentiating diabetes categories. Importantly, our results reveal that early diabetes risk stratification does not depend on any single indicator but arises from interpretable combinational patterns and interactions among a few routine clinical biomarkers.

Overall, this research demonstrates the applicability of deep learning in reliably identifying different diabetes states and progression trends under low operational burden. The lightweight, progressively regularized architecture with channel attention not only enhances accuracy and efficiency in multi-class diabetes classification but also provides an explainable, cost-effective, and clinically deployable framework. The proposed ProgMDD thus offers a practical and interpretable deep learning approach for biomarker-based disease prediction, supporting early diabetes screening and stratified intervention in real-world clinical settings. This study lays the groundwork for future integration of interpretable AI into clinical decision support systems.

## Supplementary Information

Below is the link to the electronic supplementary material.

Supplementary Material 1

Supplementary Material 2

Supplementary Material 3

## Data Availability

The original raw Iraqi diabetes dataset is publicly available at Mendeley Data [https://data.mendeley.com/datasets/wj9rwkp9c2/1].
