# Supplementary material for: An interpretable progressive residual network for automated multiclass diabetes diagnosis
Source: Sci Rep. 2026 May 4;16:20468. doi: 10.1038/s41598-026-51603-x (PMC13328672; doi:10.1038/s41598-026-51603-x)
Supplement: Supplementary file 1 — Supplementary Material 1 [file 41598_2026_51603_MOESM1_ESM.docx]

# **Supplementary Information**

**Title:** An interpretable progressive residual network for automated multiclass diabetes diagnosis

**Authors:** Huaxin Fan^1^, Zhendong Li^1^**^*^**, Ning Yan^2^, Hao Liu^1^

1 School of Information Engineering, Ningxia University, Yinchuan, China

2 Heart Centre & Department of Cardiovascular Diseases, General Hospital of Ningxia Medical University, Yinchuan, China

* Corresponding author:

E-mail: [lizhendong@nxu.edu.cn](mailto:lizhendong@nxu.edu.cn) (ZL)

# **Supplementary Note 1: Reproducibility, Experimental Settings, and Computational Efficiency**

1. Experimental Settings and Hardware Platforms

To ensure complete transparency and facilitate the reproducibility of our study, we have rigorously documented all key experimental configurations. Supplementary Table 1 summarizes the comprehensive training settings, including the precise hyperparameters, optimizer configurations, learning rate schedules, network architecture details, and regularization techniques used to train the ProgMDD model. Furthermore, Supplementary Table 2 outlines the hardware and software platforms utilized for all experiments, providing brief explanations for each component.

2. Computational Complexity and Operational Efficiency

To evaluate the clinical feasibility and deployment potential of the ProgMDD framework, especially in resource-constrained or real-time diagnostic environments, we conducted a rigorous analysis of its computational complexity and operational efficiency. The evaluation was performed on a workstation equipped with an NVIDIA RTX 4060 GPU, with a batch size of 32 across 200 training epochs.

As summarized in Supplementary Table 3, ProgMDD maintains a highly streamlined and lightweight architecture, comprising only 263.9 K trainable parameters. This translates to a minimal disk footprint of 3.11 MB when exporting the complete state_dict, which is significantly more compact than standard deep learning models used for physiological signal processing. From a computational perspective, the model requires only 0.67 M FLOPs per forward pass, representing an exceptionally low computational burden.

In terms of temporal efficiency, the entire training process on the training partition was completed in approximately 3.05 minutes, demonstrating rapid convergence and efficient optimization. Crucially, the average inference latency on the GPU was recorded at a mere 3.66 ms per sample. This near-instantaneous response time ensures that ProgMDD can provide real-time decision support in clinical workflows. These metrics collectively substantiate that the proposed architecture achieves a superior trade-off between high-precision diagnostic performance and extreme computational economy, making it an ideal candidate for integration into electronic health record (EHR) systems or portable medical devices.

Supplementary Table 1. Key experimental settings for experimentation

| Item | Setting |
| --- | --- |
| Task | 3-class classification |
| Label mapping | 0 = Non-Diabetic (N); 1 = Pre-Diabetic (P); 2 = Diabetic (Y) |
| Data split | Train/Val/Test = 70% / 10% / 20% (stratified, random_state=42) |
| Feature selection | L1-multinomial logistic (saga),  GridSearchCV (cv=5, scoring=neg_log_loss), 1-SE rule |
| Selected features | Gender, AGE, HbA1c, Chol, TG, BMI |
| Input normalization | StandardScaler (fit on train only; transform on val/test) |
| Input tensor shape | (N, 1, 6) |
| Backbone | ResNet1D + channel attention |
| Residual stages | 3 stages; blocks per stage = [2, 2, 2]; channels = [32, 64, 128] |
| Classifier head | 128 → 64 → 32 → 3 |
| Activation | ReLU |
| Weight initialization | Kaiming (He) |
| Loss | CrossEntropyLoss (label_smoothing = 0.1) |
| Optimizer | AdamW |
| Initial learning rate | 1e-3 |
| Weight decay | 5e-5 |
| Adam betas | (0.9, 0.999) |
| LR scheduler | OneCycleLR (cosine annealing) |
| Scheduler params | max_lr=9e-4; pct_start=0.15; div_factor=10;  final_div_factor=100 |
| Epochs | 200 |
| Batch size | 32 |
| DataLoader | num_workers=0; pin_memory=True |
| Dropout (input) | 0.1 |
| Dropout (residual blocks) | stage1=0.1; stage2=0.2; stage3=0.3 |
| Dropout (channel attention MLP) | 0.1 |
| Dropout (FC head) | fc1=0.2; fc2=0.1 |
| Data augmentation | Gaussian noise (σ=0.003) + random scaling (±2.5%) |
| Gradient clipping | max_norm=1.0 |
| EMA | enabled; decay=0.999; EMA weights used in validation |
| Seed control | Python/NumPy/Torch/CUDA = 42; cudnn.deterministic=True;  cudnn.benchmark=False |

Supplementary Table 2. Hardware platforms used for experimentation

| Category | Value | Brief explanation |
| --- | --- | --- |
| Operating system | Windows 11 (10.0.26100) | Operating system for training and experiment reproduction |
| CPU | AMD Ryzen 7 7735H | Data preprocessing and partial training scheduling |
| CPU cores/threads | 8 / 16 | Parallel processing capability，metrics |
| System memory  (RAM) | 16,366,567,424 bytes (~16.37 GB) | Main memory capacity |
| GPU | NVIDIA GeForce RTX 4060 Laptop GPU (1 card) | Primary computing power for deep learning training |
| CUDA availability | True | PyTorch supports CUDA acceleration |
| CUDA version | 12.1 | GPU runtime version |
| cuDNN version | 90100 (cuDNN 9.1.0) | Deep learning operator library version |
| Python | 3.12.11 (Anaconda) | Running interpreter version |
| PyTorch | 2.5.1+cu121 | Training framework |
| scikit-learn | 1.7.1 | LASSO and data partitioning |
| imbalanced-learn | 0.14.0 | Resampling |
| pandas/numpy/matplotlib | 2.3.1 / 2.2.6 / 3.10.0 | Data processing and visualization dependencies |

Supplementary Table 3. Computational Complexity and Efficiency Metrics of ProgMDD

| Metrics | value | Definition / Note |
| --- | --- | --- |
| Total Parameters | 263.9K | Total number of learnable parameters in the model. |
| Model Size (Disk) | 3.11MB | Storage space required for the exported .pth file. |
| FLOPs | 0.67 M | Floating point operations (measure of computational complexity). |
| Training Time | ~3.05min | Total time to complete training (200 epochs on GPU). |
| Inference Latency(GPU) | 3.66 ms | Average time to process a single sample on GPU. |

# **Supplementary Note 2: Dimensionality reduction algorithm selection**

The visualization of high-dimensional biomarker sequences is critical for understanding the model’s internal representations. Given the substantial overlap of the three diabetes categories in the PCA projection (Main Text Fig. 6a), a purely linear embedding (PCA) cannot separate the classes effectively.

t-SNE (Main Text Fig. 6b) addresses nonlinear structure by creating many compact local clusters that reflect nearest neighbors; however, it typically distorts the global geometry and is highly sensitive to parameters. By contrast, UMAP (Main Text Fig. 6c) is designed to preserve both local and global structure while being computationally efficient. It effectively learns a global topology of the data (preserving large-scale relationships) and, in practice, runs much faster than t-SNE on large datasets. As a result, UMAP tends to retain more of the true global structure in the embedding, so that inter-cluster distances better reflect category similarity. Importantly, in either t-SNE or UMAP plots, the cluster sizes and separations encode meaningful information about how alike or different the classes are.

Nevertheless, because both t-SNE and UMAP are stochastic and hyperparameter-dependent, using their embeddings for direct classification remains challenging. Therefore, in this work, we use UMAP as our primary visualization method (owing to its balance of local/global preservation and efficiency), with PCA and t-SNE serving as complementary baselines.
